# Evaluation of blunt pancreatic injury with contrast-enhanced ultrasonography in comparison with contrast-enhanced computed tomography

**DOI:** 10.3892/etm.2013.1009

**Published:** 2013-03-15

**Authors:** QING SONG, JIE TANG, FA-QIN LV, YAN ZHANG, ZI-YU JIAO, QIANG LIU, YU-KUN LUO

**Affiliations:** 1 Department of Ultrasound, Chinese People’s Liberation Army General Hospital, Beijing 100853;; 2 Department of Urology, The 305 Hospital of Chinese People’s Liberation Army, Beijing 100017, P.R. China

**Keywords:** pancreas, blunt injury, contrast-enhanced ultrasonography, contrast-enhanced computed tomography

## Abstract

The aim of the present study was to evaluate acute blunt pancreatic injury using contrast-enhanced ultrasonography (CEUS) in comparison with contrast-enhanced computed tomography (CECT). Superficial and deep lesions were established by blunt pancreatic injury in 40 Chinese Guangxi Bama miniature pigs. Conventional ultrasound (US), CEUS and CECT were performed to detect traumatic lesions in the pancreas. A total of 40 lesions were established, including 20 deep lesions and 20 superficial lesions. US identified 21 of the 40 lesions, including 7 of the 20 superficial and 14 of the 20 deep lesions. CEUS identified 34 of the 40 lesions, including 14 of the 20 superficial and 20 of the 20 deep lesions. CECT identified 33 of the 40 lesions, including 13 of the 20 superficial and 20 of the 20 deep lesions. The detection rate of acute blunt pancreatic injury using CEUS was significantly higher compared with that using US (85 vs. 52.5%, P<0.05), however there was no significant difference in the detection rate of pancreatic lesions between CEUS and CECT (85 vs. 82.5%, P>0.05). CEUS improves the diagnostic levels of conventional US and is comparable with CECT scans in the diagnosis of blunt pancreatic injury.

## Introduction

Blunt pancreatic injury is uncommon with a 1–5% incidence rate in blunt trauma (1). Due to its retroperitoneal location, early physical signs and symptoms are commonly non-specific. Also, blunt pancreatic injury may be overlooked in patients with extensive multi-organ trauma. A delay in diagnosis leads to complications, including pancreatitis, pancreatic abscesses/necrosis, pancreatic fistulae and pseudocysts, which result to a high mortality rate of nearly 20% (1). Therefore the prompt and accurate diagnosis of pancreatic injury is imperative (2).

Abdominal computed tomography (CT) scans are the modality of choice for evaluating stable patients with suspicious abdominal injuries. CT is efficient for the diagnosis of contusions, pancreatic disruptions and associated complications (3). The reported sensitivity and specificity rates are as high as 80% (4), with suspicious findings including peripancreatic hematomas and fluid in the lesser sac or retroperitoneum (5). CT grading of pancreatic trauma has been widely used in the clinic (6). However, CT has its disadvantages; it is expensive, not performed in real-time, requires exposure to X-ray irradiation, requires patient transfer away from the resuscitation area and in the case of contrast-enhanced CT (CECT) examinations, certain patients may suffer allergic reactions (7).

Due to being portable, less costly and usable at the bed-side, ultrasound (US) has been used for the diagnosis and follow-up of blunt abdominal trauma. However, conventional US is not able to provide an accurate evaluation of the site and the severity of pancreatic parenchymal injury and active bleeding. With the development of a second generation of sonographic contrast agents and contrast-specific technology, contrast-enhanced US (CEUS) has been shown to be an effective method in the diagnosis of hepatic, splenic and nephric trauma (8–10). Certain studies have shown significant differences in the detection rate between conventional US and CEUS (P<0.01) (11). CEUS was reported to clearly show the location, size, extension and active bleeding of trauma, which improved the detection rate from a range of 45.7–63% for conventional US to a range of 80–91.4% (9,12).

By contrast, the use of CEUS imaging to define the features of blunt pancreatic injury has not been well documented and the value of CEUS in the diagnosis of pancreatic injury has not been studied. The purpose of the present study was to present CEUS imaging findings and discuss the utility of CEUS in the evaluation of blunt pancreatic injury.

## Materials and methods

### Animal model

The experimental protocols of the present study were approved by the Institutional Animal Care and Use Committee (IACUC) of the Chinese People’s Liberation Army General Hospital and performed in accordance with the regulations for animal experiments defined by the ethics committee of The Chinese People’s Liberation Army General Hospital. In total, 40 healthy, male Chinese Guangxi Bama miniature pigs were housed individually in a windowed room at 26±1°C, with a relative humidity of 40–50%. All the animals were appropriately acclimatized and observed for a period of 1 week. Food was withdrawn the evening prior to the experiment. The pigs were anesthetized by an intra-muscular injection of pentobarbital sodium (30 mg/kg) and 1,000 ml Ringer’s solution was intravenously administered via the vena auricularis magna during the experiment. A midline laparotomy incision was made under aseptic conditions, the gastrocolic ligament was divided and the lesser sac was entered. The pancreas was then exposed and injuries were established randomly using a hemostatic pincette to crush the pancreas against the spine or posterior abdominal wall. Subsequent to establishing the injuries the incision was sutured. The present study was carried out in strict accordance with the recommendations made in the National Institutes of Health Guide for the Care and Use of Laboratory Animals (13). The protocol for animal use was reviewed and approved by the IACUC of the Chinese People’s Liberation Army General Hospital.

### Sonographic equipment and contrast agent

The baseline US and CEUS were performed with an Acuson Sequioa 512 Scanner (Siemens Medical Solutions, Mountain View, CA, USA). A 3–5 MHz transducer (4V1, Acuson) was also used. The contrast-enhanced studies were performed using the contrast-specific contrast pulse sequencing (CPS) technique at a low mechanical index (MI) of 0.15–0.17.

The contrast medium administered was SonoVue (Bracco, Milan, Italy), a second-generation blood-pool US contrast agent, which was previously reported to be useful in the detection of injuries caused by abdominal trauma (8–12). The contrast agent was injected as a bolus using a 21-gauge catheter placed in the vena auricularis magna. Immediately thereafter, a 5-ml saline solution flush (0.9% NaCl) followed (using a three-way stopcock).

### CT equipment and contrast agent

A helical Twin scan (GE Healthcare, Piscataway, NJ, USA) was used in the present study. The median slice-thickness for the contrast-enhanced images was 3 mm (range, 2–4 mm). A total of 40–50 ml IV contrast agent (iohexol, Omnipaque 300; GE Healthcare) was injected at 2.5 ml/sec. Scanning was performed from 20 sec after the onset of the contrast injection.

### Methods of examination

Conventional US (3.5–5.0 MHz, 30–50 mm deep) was performed within 30 min of the establishment of the pancreatic trauma. The focus was set at the deeper level of the lesion. The location, size, extent and characteristics of the laceration were recorded. Depending on the conventional US findings, CEUS was performed as a focused examination of the area of interest. Subsequent to changing to CPS at a low MI (0.15–0.17), a contrast agent bolus of 1.2 ml was injected into the vena auricularis magna. The region of interest was then slowly and continuously scanned for up to 2 min, until the enhancing effect began to disappear. At the moment of injection, a timer was pushed and video archiving was started. All images were analyzed by two sonographers, each with a minimum of 5 years CEUS experience, who were blinded to the establishment of the traumatic models.

A CECT scan was performed within 30 min subsequent to the CEUS examination. The sweep range ran from the diaphragmatic muscle to the inferior margin of the kidney. CT images were reviewed by two experienced radiologists with 5 and 7 years of experience in abdominal imaging. They were kept blinded to the results obtained from the US and CEUS imaging modalities and other techniques.

Following the examination, a laparotomy was performed in each pig by a surgeon who was not involved in the process of injury establishment and examination. The pancreatic duct injuries were identified by the injection of methylene blue through the duodenal papilla. All the lesions were then classified as deep or superficial. Deep lesions were defined as hematomas or lacerations that were ≥50% of the thickness of the pancreas. Superficial lesions were described as hematomas or lacerations that were <50% of the thickness of the pancreas.

### Statistical analysis

Measurements are presented as mean ± SD. Differences between group means were compared by a Student’s t-test. Ratio proportions were compared using a Chi-square test with continuity corrections or a Fisher’s exact test when appropriate. The level of statistical significance was set at P<0.05. Two-sided significance tests were used throughout.

## Results

### Injuries

In total, forty male pigs with a mean weight of 22.2±1.7 kg (range, 20–25 kg) were used. A total of forty injury sites were established, including twenty deep sites with an average depth of 1.5±0.4 cm (range, 0.9–2.2 cm) and twenty superficial sites with an average depth of 0.5±0.2 cm (range, 0.2–0.9 cm).

Of the twenty deep lesions, eighteen lesions with main pancreatic duct injuries (MPD^+^) and two lesions without main pancreatic duct injuries (MPD^−^) were identified in the laparotomy. Of the twenty superficial lesions, one MPD+ lesion was detected.

The levels of serum amylase and lipase were observed to increase during the initial one hour period following pancreatic trauma (Tables I and II). However, there were no significant differences between the levels in the superficial and deep lesions (P>0.05).

### Sonographic findings

In the contrast enhancement analysis, two dynamic phases were considered: the arterial phase, 10–30 sec subsequent to injection; and the venous phase, 30–120 sec subsequent to injection.

**Figure 1:**
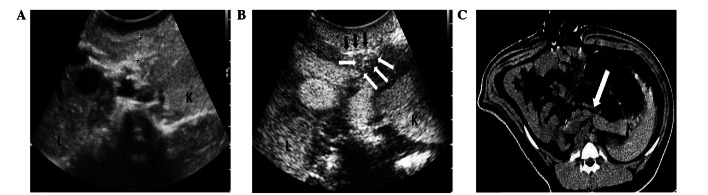
Images of a deep laceration. (A) A deep laceration of the pancreas was not clearly shown in the US image. The parenchyma shown between the plus signs was inhomogeneous. (B) The laceration (white arrow) was identified as inhomogeneous with high enhancement by CEUS. The accumulation of the contrast agent was shown as hyperechoic in the interstice between the pancreas and bursa omentalis (black arrows). (C) The laceration was hypointensive in the CECT image. K, kidney; L, liver; US, ultrasound; CEUS, contrast-enhanced ultrasonography; CECT, contrast-enhanced computed tomography.

**Figure 2:**
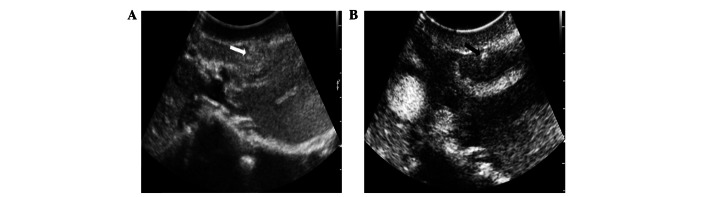
Images of a superficial laceration. (A) The parenchyma of the pancreas (white arrow) was inhomogeneous in the US image. (B) A superficial laceration of the pancreas (black arrow) was clearly shown by CEUS. The laceration was highly enhanced in the CEUS image due to active bleeding. US, ultrasound; CEUS, contrast-enhanced ultrasonography.

A total of twenty-one lesions were detected using conventional US (Figs. 1 and 2), while nineteen lesions remained undetected. The detection rate of conventional US in pancreatic trauma was 52.5% (Table III). The pancreatic lacerations appeared as slit- or stripe-shaped hyperechoic, hypoechoic or anechoic areas on the baseline US image. The pancreatic contusions appeared as lamellar hyperechoic or hypoechoic areas with unclear margins in the parenchyma on conventional US images.

Following the injection of the contrast agent, 34 lesions were detected and 6 lesions remained undetected by CEUS. The detection rate of CEUS in pancreatic trauma was 85%, which was significantly higher than that of conventional US, (P<0.05; Table III). The six lesions that were not revealed by CEUS were MPD^−^ lesions. The pancreatic lacerations showed no enhancement in the two phases, however a high enhancement was observed in the two phases due to active bleeding (Figs. 1 and 2). The pancreatic contusion showed low enhancement in the two phases. The margins of the pancreatic injuries were clearly observed compared with the normal parenchyma by CEUS.

Peripancreatic fluid collections were identified in all pigs by US and CEUS.

### CECT findings

CECT was performed in all pigs; 33 lesions were diagnosed as pancreatic injuries and 7 were not. The detection rate of CECT in pancreatic trauma was 82.5% (Table IV). A total of twenty deep lesions and thirteen superficial lesions were identified by CECT imaging. All lesions that were not revealed using CECT were MPD^−^ lesions. All pigs with hemoperitoneum were successfully identified by CECT imaging.

The lacerations were visible as low-attenuation lines oriented perpendicularly to the long axis of the pancreas (Fig. 1). The diagnosis of a pancreatic parenchymal fracture by CECT was based on the visualization of a clear separation of the pancreas. Certain fractures or lacerations of the pancreas produced little separation of the fragmented tissue and showed no enhancement in the two phases. The contusions appeared as diffuse areas of low attenuation or focal areas of low attenuation within the normal parenchyma.

There was no significant difference in the detection rate of pancreatic lesions between CEUS and CECT (P>0.05; Table IV). The main CEUS and CECT findings from the forty pigs with blunt pancreatic injuries are presented in Table V.

## Discussion

Injuries to the pancreas are rare. The initial signs and symptoms of pancreatic injury may be subtle and laboratory findings of pancreatic injury are nonspecific, which may consequently lead to delayed or missed diagnoses and substantial morbidity and mortality rates. Matsuno *et al*(14) reported that a delayed serum amylase and lipase measurement may be useful to detect pancreatic injuries. In the data from the present study, elevated serum amylase and lipase levels were observed in 36 and 35 pigs, respectively. This indicated that amylase and lipase measurements are useful to detect pancreatic injuries, but that the elevated level is not correlated with the severity of the injury. This result conforms with the findings of other studies (15,16).

Blunt pancreatic injuries are usually caused by a direct force applied across the upper abdomen by a seat belt, the handlebar of a bicycle or motorcycle or by a steering wheel, which compresses the pancreas against the spine (17). Therefore, a pancreatic parenchymal compression method was adopted to establish the pancreatic injury model in the present study and to imitate the mechanism by which blunt pancreatic injuries usually occur.

In the early phase of the trauma (72 h post-injury), the identification of the pancreatic trauma was difficult. Although CT has advantages in the diagnosis of pancreatic trauma (18,19), it is not available to the first choice for hemodynamically unstable patients. Due to its characteristics of being noninvasive, versatile, easily accessible and less costly, US plays a significant role in assessing pancreatic trauma in the early phase. US effectively complements CT as an auxiliary examination for patients who are unstable or allergic to diodone.

Nevertheless, the sensitivity of conventional US in the diagnosis of pancreatic trauma is reported to be low (45.7–63%) (9–12), which conforms with the results of the present study. CEUS is more sensitive than conventional US in identifying the characteristics of the injury, including the size and the tear thickness (20). In the present study, the sites of laceration often appeared with low enhancement. Occasionally the sites appeared with high enhancement, due to the presence of a hemorrhage caused by injury to the small peripancreatic vessels, which was always associated with extra-pancreatic fluid collections. When the fracture was occupied by fluid, no enhancement was visible in the CEUS images. These sonographic depictions were similar to those in the CECT images. These results suggested that CEUS is almost as sensitive as CECT for depicting blunt pancreatic injuries.

The detection of pancreatic duct injuries is critical to the subsequent treatment of the patient. Unfortunately, the integrity of the pancreatic duct is difficult to determine by CT and US. Therefore, the depth of the pancreatic injury was used in the present study, instead of a direct description, to determine the injury to the pancreatic duct (21). Wong *et al*(4) suggested that a CT finding of a lesion of >50% of the thickness of the pancreas indicated a likely disruption to the pancreatic duct. This theory is in line with the findings of the present study. In total, 90% of the deep lesions were confirmed as MPD^+^ and 95% of the superficial lesions were confirmed as MPD^−^. Pancreatic trauma with MPD^+^ requires surgical treatment and the results of CEUS and CECT imaging may help decide what treatment is required. It is important to separate patients into two groups, those who require immediate surgery and those who require non-surgical observation. The American Association for the Surgery of Trauma Organ Injury Scale for pancreatic injury was not used in the present study, as only a few surgical or radiological studies remarked upon the integrity of the pancreatic duct (21,22).

However, false negative results were observed in CECT and CEUS. This may be associated with the depth or appearance of the lesion. In total, 7 lesions were not identified by CECT and 6 were not identified by CEUS. Lesions are missed by CECT when the depth is less than the slice thickness. This problem may be avoided by using CEUS. The minimum depth shown by CEUS is 2 mm. However, pancreatic fracture lines are not easily detected by CEUS when the separation of the fractured pancreatic fragments is minimal or nonexistent (23). Overestimation on CEUS may occur as deep lacerations are occasionally not associated with a disruption of the main duct and transections may merely disrupt the minor duct, as in the present experiment.

In summary, CEUS is able to successfully diagnose the majority of acute blunt pancreatic injuries and exactly evaluate the extent of the trauma. However, a normal appearance in CEUS is not able to exclude pancreatic injury. Repeated physical examinations and further imaging studies may aid the identification of acute pancreatic trauma and its mechanisms.

## Figures and Tables

**Table I:** Serum amylase mean values during the initial 1 h period following pancreatic trauma.

Lesions	Amylase (IU/l)	
	Pre-injury	1st hour
Total	1373±349	2845±520
Deep	1295±370	2874±480^a^
Superficial	1451±316	2714±608^a^

a P=0.3622 vs. superficial group at 1st hour.

**Table II:** Serum lipase mean values during the initial 1 h period following pancreatic trauma.

Lesions	Lipase (IU/l)	
	Pre-injury	1st hour
Total	1088±246	1646±417
Deep	1080±235	1591±281^a^
Superficial	1095±262	1705±512^a^

a P=0.3861 vs. superficial group at 1st hour.

**Table III:** Comparison of the detection rate between US and CEUS in the diagnosis of pancreatic lesions.

Group	Lesions		
	Superficial	Deep	Total
US, n (%)	7 (35)	14 (70)	21 (52.5)
CEUS, n (%)	14 (70)	20 (100)	34 (85)
P-value	0.0418	0.0202	0.0038

US, ultrasonography; CEUS, contrast-enhanced ultrasonography.

**Table IV:** Comparison of the detection rate in the diagnosis of pancreatic lesions between CEUS and CECT.

Group	Lesions		
	Superficial	Deep	Total
CECT, n (%)	13 (65)	20 (100)	33 (82.5)
CEUS, n (%)	14 (70)	20 (100)	34 (85)
P-value	1.0000	-	1.0000

CEUS, contrast-enhanced ultrasonography; CECT, contrast-enhanced computed tomography.

**Table V:** CEUS and CECT findings in the forty pigs with blunt pancreatic injuries.

Main signs	CEUS	CECT
Fracture of the pancreas	5	5
Pancreatic laceration	28	29
Contusion	15	13
Focal or diffuse pancreatic enlargement	29	29
Peripancreatic fluid collection	40	40

CEUS, contrast-enhanced ultrasonography; CECT, contrast-enhanced computed tomography.
